# Precision in Delivery, Variability in Response: A Multiscale Mechanistic Framework for Neuronavigated Transcranial Magnetic Stimulation

**DOI:** 10.3390/brainsci16090901

**Published:** 2026-08-23

**Authors:** Marcin Karol Setlak, Bartłomiej Błaszczyk, Maciej Wojtacha, Adam Rudnik

**Affiliations:** Department of Neurosurgery, University Clinical Center, Faculty of Medical Sciences in Katowice, Medical University of Silesia, 40-752 Katowice, Poland

**Keywords:** neuronavigated transcranial magnetic stimulation, repetitive transcranial magnetic stimulation, cortical excitability, cellular mechanisms, synaptic plasticity, electric-field modeling, brain connectivity, network neuroscience

## Abstract

**Highlights:**

**What are the main findings?**

- Neuronavigation improves the spatial and orientational reproducibility of neuronavigated transcranial magnetic stimulation (nTMS) delivery but does not, by itself, confirm biological target engagement or response.
- Response variability arises across biophysical, cellular, plasticity, and network levels, allowing uncertainty to propagate through the stimulation-response cascade.

**What are the implications of the main findings?**

- Precision nTMS requires independent validation of modeled exposure, physiological and network engagement, and durable functional or clinical outcomes.
- Adaptive targeting and dosing should be guided by multimodal measurements rather than geometric accuracy alone.

**Abstract:**

Background/Objectives: Transcranial magnetic stimulation (TMS) initiates a cascade from intracranial electric-field exposure through neural recruitment and plasticity to distributed network responses. Neuronavigation improves the geometric reproducibility of delivery but does not guarantee equivalent cortical exposure or target engagement. This narrative review integrates these levels within an operational framework for precision TMS. Methods: Six domain-specific PubMed searches covering 1 January 1985 to 31 July 2026 were supplemented by Google Scholar and citation tracking. A documented rerun on 17 August 2026 yielded 6430 records (5617 unique after cross-query deduplication). Evidence was synthesized narratively; no quantitative synthesis or formal risk-of-bias assessment was performed. Results: Neuronavigation improves geometric precision by stabilizing target definition and coil pose, whereas individualized electric-field models estimate intracranial exposure. Neither establishes biological precision, which also depends on neuronal orientation, brain state, circuit architecture, medication, and behavior. Motor-system measures are not validated as universal biomarkers for nonmotor cortex, and no single validated biomarker captures TMS-induced plasticity. Convergent, controlled multimodal evidence may strengthen inference about target engagement; adaptive and closed-loop approaches remain experimental. Conclusions: Geometric delivery, modeled exposure, biological engagement, and durable functional or clinical benefit require separate validation. Spatial accuracy alone does not establish clinical value.

## 1. Introduction

Transcranial magnetic stimulation (TMS) uses electromagnetic induction to probe and modulate human brain function [1,2]. Since Barker et al. first elicited motor responses in 1985, TMS has developed into a tool for functional mapping, assessment of cortical excitability and connectivity, and therapeutic neuromodulation [1,2]. A rapidly changing current in the stimulation coil generates a magnetic field that induces an electric field in brain tissue. Neural recruitment is shaped not only by stimulator output but also by pulse waveform, coil geometry and orientation, coil-to-cortex distance, tissue conductivity, cortical folding, axonal geometry, and the state of the stimulated system [2,3]. Magnetic resonance imaging (MRI)-based neuronavigation enabled TMS to be delivered while tracking coil position and orientation relative to individual anatomy. In this review, neuronavigated TMS (nTMS) refers to this image-guided mode of delivery. Neuronavigation improves spatial control and reproducibility, but it neither changes the stimulation waveform nor introduces a separate biological mechanism [4,5,6].

The biological effects of TMS extend beyond the brief induced electric field. A single pulse recruits suitably oriented axonal elements and, through them, local excitatory and inhibitory circuits [3,7,8]. Repeated stimulation may produce long-term potentiation (LTP)-like or long-term depression (LTD)-like after-effects, metaplasticity, and activity-dependent molecular or structural change [7,8,9]. These responses can propagate through connected networks [10], but their direction and magnitude depend on pulse pattern, intensity, total pulse count, temporal spacing, baseline excitability, ongoing brain state, anatomy, medication, and behavior [7,9]. Frequency-based labels are therefore useful protocol descriptors, not universal mechanistic rules [5,11,12].

Evidence for this cascade is distributed across computational biophysics, cellular and animal models, human neurophysiology, and neuroimaging [3,8,9,10,13]. In nTMS, coil pose interacts with cortical geometry and tissue heterogeneity to shape the strength, direction, and distribution of the electric field reaching excitable elements [3,4,13,14]. Neuronavigation therefore controls the geometric interface between an intended target and the delivered physical stimulus; it does not control downstream state- or network-dependent variability.

This narrative review integrates biophysical, cellular, synaptic, molecular, and network evidence into a multiscale framework for nTMS. It distinguishes geometric, biophysical, and biological precision; examines individualized modeling, mechanistic biomarkers, protocol dependence, and adaptive stimulation; and identifies the evidence required to link reproducible delivery with a durable functional or clinical response.

## 2. Materials and Methods

### 2.1. Review Design and Scope

This narrative review was prepared with reference to relevant principles of the Scale for the Assessment of Narrative Review Articles (SANRA) [15]. It was designed to integrate heterogeneous mechanistic evidence rather than estimate clinical efficacy or perform a meta-analysis. The scope included the generation and intracranial distribution of TMS-induced electric fields; recruitment of neural elements and local microcircuits; synaptic, molecular, and glial mechanisms of plasticity; propagation through distributed brain networks; and the influence of neuronavigation and individualized electric-field modeling on target engagement. Because neuronavigation constrains stimulus delivery rather than introducing a separate biological mechanism, evidence from conventional single-pulse, paired-pulse, repetitive, and patterned TMS was included whenever it informed the interpretation of nTMS.

### 2.2. Information Sources and Study Selection

Literature published from 1 January 1985 to 31 July 2026 was identified through domain-specific PubMed searches, supplemented by Google Scholar and backward and forward citation tracking. The PubMed searches were rerun and documented on 17 August 2026. Six searches addressed the following: (i) biophysics and electric-field distribution; (ii) neural recruitment and local microcircuits; (iii) synaptic, molecular, and glial mechanisms; (iv) network-level effects, TMS combined with electroencephalography (TMS–EEG), and neuroimaging; (v) neuronavigation, targeting, and individualized modeling; and (vi) variability, biomarkers, and adaptive stimulation. Complete search strings and domain-specific retrieval counts are provided in Supplementary Table S1. Across the six searches, PubMed returned 6430 records before cross-query deduplication and 5617 unique PubMed records. Google Scholar and citation chaining were used to identify foundational studies and newly linked sources; because their rankings and result counts are dynamic, they were not treated as countable screening sets.

Eligible sources included English-language human, animal, in vitro, and computational studies that addressed at least one level of the proposed framework. Priority was given to primary studies providing direct mechanistic evidence. Reviews, consensus statements, methodological papers, and guidelines were used to contextualize original findings, while clinical studies were included when they reported mechanistic, neurophysiological, neuroimaging, or targeting-related outcomes. Selected recent preprints were considered only when rapidly developing questions were not adequately represented in the peer-reviewed literature; their findings were identified as preliminary and were not used as the sole support for major conclusions. Studies limited to clinical efficacy, tolerability, or safety, together with conference abstracts, protocols, duplicate reports, and publications lacking substantive mechanistic information, were excluded.

The first author conducted the searches and initial eligibility assessment, and the coauthors critically reviewed the selected evidence and final synthesis. Records were not managed as a closed systematic-review cohort. Selection was iterative and purposive, prioritizing direct mechanistic evidence, replication, and convergence across complementary methods. The principal exclusion categories are stated above. Because record-level inclusion decisions and, where applicable, reasons for exclusion were not logged prospectively, an accurate Preferred Reporting Items for Systematic Reviews and Meta-Analyses (PRISMA)-style flow diagram and exclusion counts cannot be reconstructed without retrospective assumptions. We therefore report this limitation rather than provide retrospective estimates. The final synthesis comprises 131 cited sources.

### 2.3. Evidence-Type Stratification

To make the source and limits of each inference explicit, studies were grouped into four non-equivalent categories before findings were integrated across mechanistic levels.

#### 2.3.1. Computational Evidence

Computational studies estimated how individual anatomy and coil configuration shape intracranial exposure. Subject-specific models showed that tissue boundaries and gyral geometry alter field magnitude and direction [13,14], while multiscale neural models generated hypotheses about orientation-dependent recruitment [3]. These studies support biophysical and hypothesis-generating cellular inferences, not direct claims of target engagement or clinical efficacy [16].

#### 2.3.2. In Vitro Evidence

In vitro and organotypic studies directly examined synaptic efficacy, receptor-dependent plasticity, intracellular signaling, gene expression, and glial responses under controlled conditions [8,9]. The included experiments report persistent synaptic and structural changes after repeated magnetic stimulation (Section 6), but their anatomy, scale, and exposure differ from those of the intact human neocortex.

#### 2.3.3. Animal Evidence

Animal studies linked cellular and molecular responses to changes in intact circuits, glia, structure, and behavior [8,9]. In the included corpus, these effects depended on protocol, species, disease model, and anesthesia (Section 6 and Section 7); they were therefore treated as preclinical evidence rather than assumed to be equivalent to human nTMS.

#### 2.3.4. Human Evidence

Human studies demonstrated current-direction-dependent corticospinal recruitment [3], improved coil-pose reproducibility with neuronavigation [4], state-dependent physiological responses [9], and network propagation [10]. Their relevance to clinical nTMS varied because many studies involved small samples, healthy volunteers, motor-cortex paradigms, or short-term surrogate outcomes (Section 4, Section 5, Section 6, Section 7, Section 8, Section 9, Section 10 and Section 11).

### 2.4. Narrative Synthesis and Appraisal

Within each category, studies were appraised qualitatively according to reported methodological safeguards, directness, replication, consistency with complementary methods, and relevance to human nTMS. Findings were then integrated along the proposed sequence from physical delivery to neural recruitment, local circuit responses, plasticity, and distributed network effects. Discrepant results were considered in relation to species, preparation, brain state, target, stimulation parameters, coil orientation, and measurement technique. Formal study-level risk-of-bias tools were not prespecified for this narrative review. The review did not include duplicate screening, prospective protocol registration, or quantitative synthesis. This qualitative appraisal does not eliminate author judgment and remains vulnerable to selection and interpretive bias; these limitations are detailed in Section 12.1.

## 3. A Multiscale Framework for nTMS Mechanisms

Precision nTMS comprises distinct levels. Geometric precision concerns reproducible target definition and coil pose; biophysical precision concerns the individualized magnitude, direction, and distribution of the intracranial electric field; and biological precision concerns reproducible recruitment of the intended neural elements and circuits. Durable functional or clinical benefit is a separate endpoint. Programmed parameters, physical exposure, engagement, and outcome are related but not interchangeable [3,4,13,14,17].

**Figure 1:**
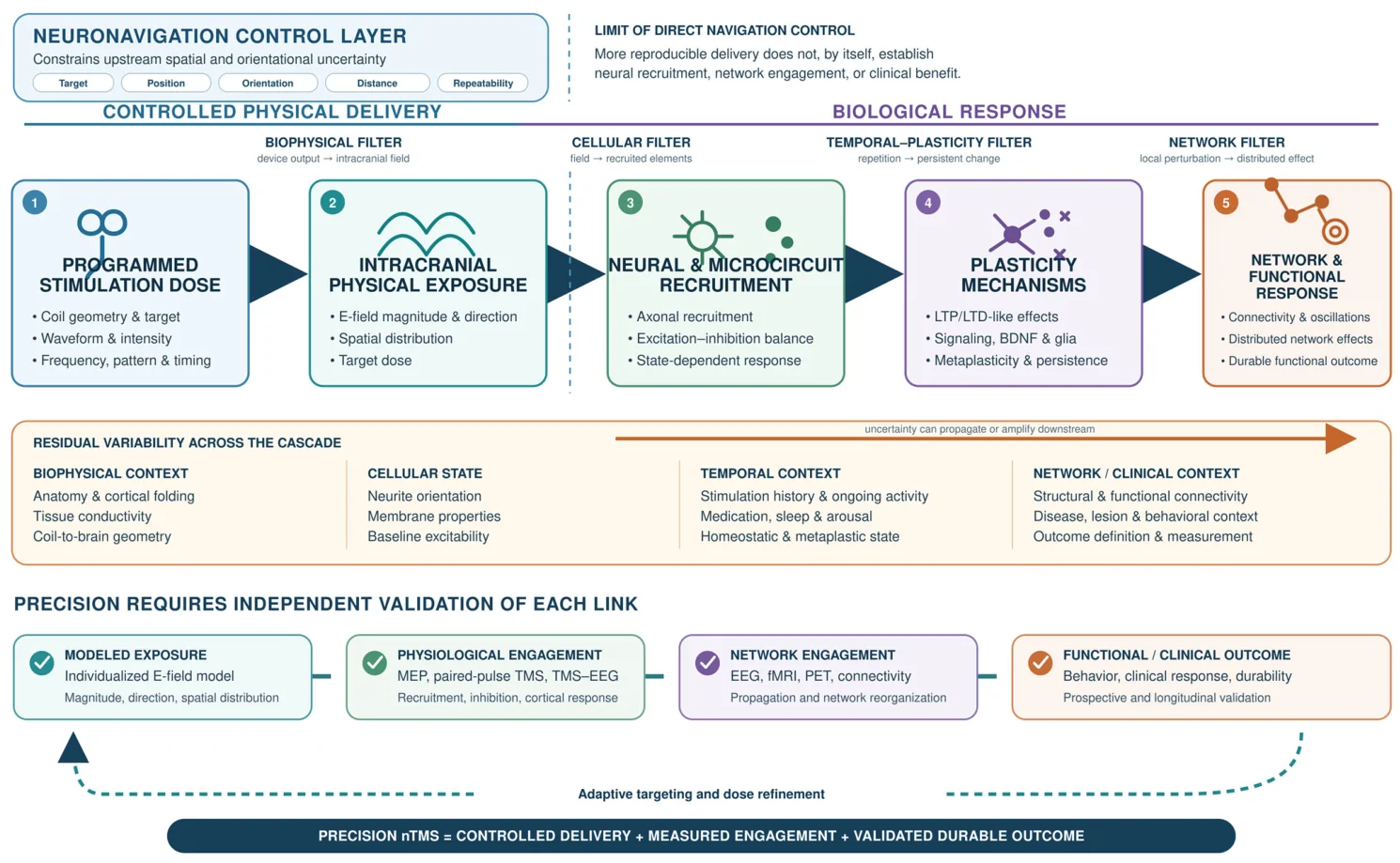
Multiscale mechanistic framework for precision neuronavigated transcranial magnetic stimulation. Programmed stimulation parameters are transformed into intracranial physical exposure through a biophysical filter shaped by coil–brain geometry, individual anatomy, cortical folding, and tissue conductivity. The cellular filter determines the recruitment of axons and local microcircuits; repeated activation is integrated by temporal-plasticity mechanisms; and the network filter governs whether the perturbation remains local or propagates through distributed circuits to produce a functional response. Neuronavigation constrains upstream spatial and orientational uncertainty but does not, by itself, establish downstream biological engagement. Precision nTMS therefore requires independent validation of modeled exposure, physiological target engagement, network engagement, and durable functional or clinical outcomes, with feedback enabling adaptive refinement of targeting and stimulation parameters. Abbreviations: BDNF, brain-derived neurotrophic factor; E-field, electric field; EEG, electroencephalography; fMRI, functional magnetic resonance imaging; LTP/LTD, long-term potentiation/depression; MEP, motor evoked potential; nTMS, neuronavigated transcranial magnetic stimulation; PET, positron emission tomography; TMS–EEG, concurrent transcranial magnetic stimulation and electroencephalography.

**Figure 2:**
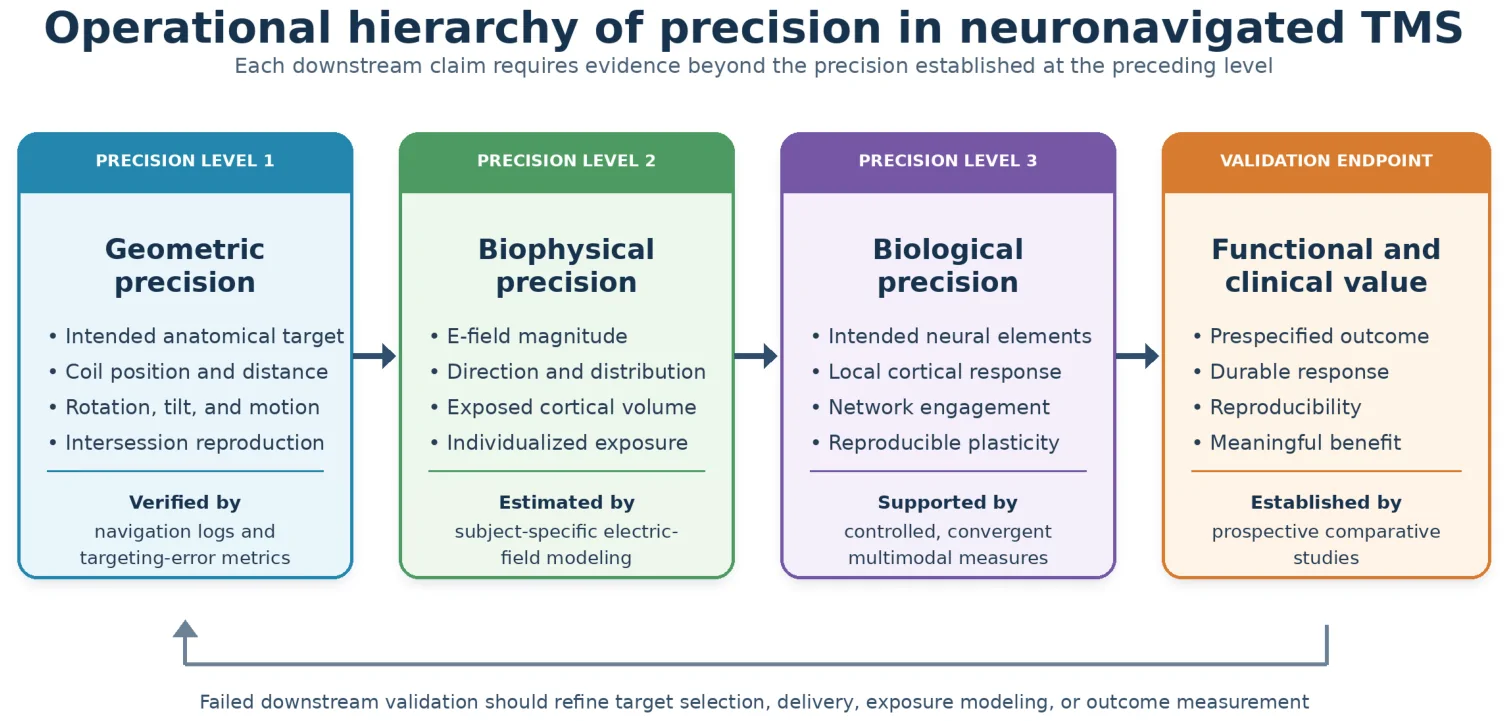
Operational hierarchy of precision in neuronavigated transcranial magnetic stimulation. Geometric precision denotes reproducible target definition and coil pose; biophysical precision denotes reproducible individualized intracranial electric-field exposure; and biological precision denotes reproducible engagement of the intended neural elements, circuit, and downstream response. Functional or clinical value is a separate validation endpoint. Evidence at one level does not establish the next, and failed downstream validation should prompt refinement of target selection, delivery, exposure modeling, or outcome measurement. Abbreviations: E-field, electric field; nTMS, neuronavigated transcranial magnetic stimulation.

Four filters connect these levels: biophysical (device output to intracranial field), cellular (field to neural and microcircuit recruitment), temporal-plasticity (integration with stimulation history and ongoing activity), and network (local response to distributed propagation) [3,8,9,10,13,14,17,18]. Neuronavigation mainly constrains upstream geometry; later links require independent physiological, network, and outcome validation. Figure 1 and Figure 2 summarize the cascade and operational hierarchy [5,12,16].

## 4. Biophysical Foundations of Neuronavigated TMS

### 4.1. Electromagnetic Induction and the Induced Electric Field

A TMS pulse is produced by rapid coil-current discharge. The resulting time-varying magnetic field penetrates the scalp and skull and induces an electric field in conductive brain tissue [17,19]. Under the quasistatic approximation, its temporal profile is governed mainly by the rate of change of coil current. The nonuniform intracranial field contains a primary component generated by the changing magnetic vector potential and a secondary component created by charge accumulation at conductivity boundaries [13,19]. Cerebrospinal fluid spaces, gray–white matter boundaries, skull geometry, and cortical folding reshape field magnitude and direction [3,13]. Recruitment depends on field strength, orientation, and spatial variation relative to excitable structures [3]; nominal stimulator output and scalp coordinates therefore do not fully define the cortical stimulus.

### 4.2. Coil Geometry, Orientation, and Pulse Waveform

Coil geometry determines the exposure pattern. Circular coils generate broad fields, whereas figure-of-eight coils provide greater focality at superficial targets [20]. Increasing depth generally enlarges the exposed superficial volume, as shown in comparisons of conventional and deep-TMS coil designs [20,21]. Coil design must therefore balance depth, focality, energy demand, heating, and practical constraints [22]. A coordinate beneath the coil should not be equated with the stimulated volume; estimating that volume requires an appropriate coil model and individualized electric-field calculation [4,20,21,22].

Coil rotation changes electric-field direction relative to gyral and sulcal anatomy and can shift the location and magnitude of maximal cortical exposure [23]. The optimal orientation varies across regions and individuals because it depends on local geometry and the alignment of excitable neural structures [23,24]. Pulse width and waveform add selectivity: pulse width influences the cortical strength–duration relationship, motor threshold, and input–output curve, while monophasic and biphasic pulses may recruit partly different populations [25,26]. Pulse shape can also alter neuromodulatory after-effects [27]. A recent systematic review of nine concurrent TMS–EEG studies found current direction to be especially influential for early TMS-evoked potentials (<50 ms), with waveform-dependent effects also extending to oscillations and functional connectivity [28]. Coil pose, current direction, pulse width, waveform, intensity, frequency, pulse number, and temporal structure should therefore be reported as programmed stimulation parameters; none alone constitutes a biological dose.

### 4.3. Individual Anatomy and Tissue Properties

Intracranial electric-field exposure can differ substantially between individuals even when coil type, position, orientation, waveform, and stimulator output are held constant. Coil-to-cortex distance is a major determinant of effective stimulation: greater separation requires higher output to produce comparable cortical activation [29]. Skull geometry, cerebrospinal fluid distribution, cortical folding, and tissue interfaces further modify field magnitude and spatial distribution [13,14,30,31]. In simulations using 50 anatomically distinct head models, greater brain–scalp distance and cerebrospinal fluid thickness were associated with weaker and more dispersed fields [31]. Physiological validation of subject-specific finite-element models also supports the value of individual anatomy for predicting cortical exposure [32]. Anatomical neuronavigation can reproduce coil placement without ensuring equivalent intracranial exposure across individuals.

Pathological anatomy adds uncertainty by altering tissue geometry and conductivity. Models of chronic and pediatric stroke show that infarct cavities, ventricular enlargement, and atrophy can create local hotspots and distort field topology [33,34]. Effects depend on lesion composition, location, surrounding anatomy, and coil configuration. In six patients with tumors near the precentral gyrus, subject-specific predictions overlapped by up to 80% with areas identified by intraoperative direct electrical stimulation [35], providing small-cohort proof of concept. Edema, postoperative cavities, skull defects, and cranial reconstruction should likewise be represented when they lie near the target; coordinate-based navigation alone may otherwise mischaracterize the exposed tissue.

### 4.4. Electric-Field Modeling and Individualized Dosimetry

Computational electric-field modeling links programmed stimulation parameters to intracranial exposure. A subject-specific workflow segments structural MRI into relevant tissue compartments, assigns conductivity values, and combines the resulting mesh with the coil model and recorded pose [36,37]. Numerical methods, usually finite-element methods, then estimate field magnitude and direction within predefined cortical targets [36,37,38]. The result is a biophysical exposure estimate, not a map of neuronal activation, which also depends on morphology, axonal orientation, membrane properties, and waveform [3,17,38]. Percentages of maximum stimulator output or motor threshold standardize device settings without ensuring equivalent cortical exposure, especially at nonmotor targets [16].

Electric-field-based dosing attempts to standardize modeled intracranial exposure. One approach estimates the cortical field associated with an individual motor threshold and adjusts intensity at a nonmotor target toward that value [39,40]. This can account for regional differences in coil-to-cortex distance, folding, and tissue composition, but the optimization objective is not neutral. Maximizing total field magnitude, a normal or tangential component, spatial extent, orientation, or a mean, maximum, or percentile value within a region of interest can select different coil configurations [37,40,41]. An optimized field metric is therefore not equivalent to optimized neuronal recruitment or clinical response. Reproducible use requires reporting the imaging and segmentation pipeline, conductivities, coil model and recorded pose, field component, region-of-interest definition, summary metric, and reference exposure [16].

## 5. Cellular and Microcircuit Mechanisms

### 5.1. Membrane Polarization and the Sites of Neural Excitation

At the cellular level, the induced field polarizes neuronal processes according to their alignment and the field variation along their trajectories [3,42]. Axons are generally more excitable than somata or dendrites, particularly at terminals, branches, bends, diameter changes, and sharp field gradients [42,43]. Early models favored the axon initial segment, bends, or the gray–white matter boundary [42,44], whereas models with richer axonal arborizations often identified intracortical terminals near gyral crowns and lips [43,45]. Somata and dendrites may undergo subthreshold polarization but are unlikely to be the principal initiation sites under conventional conditions [3,42,43]. TMS is therefore best viewed as an axonal stimulus whose site of excitation depends on axonal geometry, myelination, field direction, waveform, and cortical anatomy; initiated spikes can then recruit local and distant neurons synaptically [3].

Model disagreement largely reflects assumptions about microscopic anatomy and axonal biophysics. Activation cannot be predicted from field magnitude at the soma or a single cortical point; it depends on exposure along the excitable cable [46,47]. Field rotation can therefore change the branch that reaches threshold even when overall magnitude is unchanged [45,46]. Predictions remain sensitive to axonal diameter, myelination, internodal and branch geometry, and the completeness of reconstructed collaterals [43,45,46]. Models with limited collaterals tend to favor initial segments, bends, or the gray–white matter transition, whereas models with myelinated terminal branches more often favor intracortical terminals [42,43,44,45]. A histology-informed preprint instead reported high thresholds at incompletely myelinated cortical terminals and identified myelinated bends entering superficial white matter as plausible targets [48]. The first compartment activated by TMS thus remains unresolved. Neuronavigation constrains macroscopic exposure, not the microscopic initiation site, although small orientation changes may alter the recruited axonal subset.

### 5.2. Direct and Transsynaptic Recruitment of Cortical Microcircuits

After an action potential is initiated, the response is shaped by propagation through local and long-range circuits. Cervical epidural recordings distinguish an early direct wave (D-wave) from later indirect waves (I-waves) [49,50]. D-waves mainly reflect direct corticospinal-axon excitation, whereas I-waves reflect synchronized transsynaptic recruitment of corticospinal neurons through cortical interneuronal networks [50]. Their relative contribution depends on intensity, waveform, current direction, and anatomy [49,50,51,52]. The motor-evoked potential (MEP) integrates these cortical events with corticospinal transmission, spinal excitability, neuromuscular propagation, and recording conditions. It is not a direct measure of local membrane depolarization or activated cortical volume. Because this evidence derives predominantly from the motor cortex, motor threshold and MEP-based measures should not be extrapolated automatically to nonmotor targets as universal measures of cortical excitability.

I-wave periodicity is thought to emerge from recurrent interactions among supragranular excitatory neurons, inhibitory interneurons, and layer V corticospinal neurons, but the cellular composition remains unresolved [53]. Posterior-to-anterior stimulation preferentially recruits a low-threshold I1 pathway, whereas anterior-to-posterior stimulation favors later, more dispersed inputs [51,52,53]. Afferent inhibition and paired-pulse facilitation further support partly distinct early and late circuits [52,53,54]. Robot-assisted neuronavigated mapping also found spatially shifted representations for short-interval intracortical facilitation relative to single-pulse motor maps [54]. Reproducible microcircuit engagement therefore requires stable orientation, tilt, and current direction in addition to anatomical localization.

### 5.3. Excitatory–Inhibitory Balance Within Cortical Microcircuits

Paired-pulse paradigms probe the simultaneous recruitment of excitatory and inhibitory circuits. A subthreshold conditioning pulse produces short-interval intracortical inhibition (SICI) at approximately 1–5 ms and commonly reveals intracortical facilitation (ICF) at 6–20 ms [55]. Lorazepam enhances SICI and suppresses facilitation [56], whereas the NMDA-receptor antagonist dextromethorphan suppresses ICF [57]. At 50–200 ms, long-interval intracortical inhibition (LICI) is associated mainly with slower GABAB-receptor signaling and is enhanced by baclofen [58]. These are systems-level probes, not receptor-specific assays; their magnitude depends on pulse intensities, current direction, recruited populations, and interactions among circuits.

Inhibitory control is nonlinear. SICI and LICI involve partly distinct populations, and presynaptic GABAB activation can attenuate a GABAA-related SICI response while strengthening other inhibition [58]. SICI also preferentially affects later I-wave pathways and can reduce repetitive spinal motoneuron discharges [59]. Conversely, overlapping short-interval intracortical facilitation can mask SICI and mimic reduced inhibition [60]. Changes in SICI, ICF, or LICI therefore cannot be translated directly into cortical GABA or glutamate concentrations, nor can human TMS reliably assign them to individual interneuron classes [3,53]. Increasing intensity may change circuit composition rather than simply amplify one response; neuronavigation stabilizes the physical input, not this biological input–output relationship.

### 5.4. State Dependency and Nonlinear Recruitment

The response to TMS depends on circuit state at the moment of stimulation. Voluntary contraction increases descending I-waves and motor-evoked potentials [50]. Real-time electroencephalography (EEG)-triggered stimulation likewise produced LTP-like facilitation during a high-excitability phase of the sensorimotor μ-rhythm but not during a low-excitability phase [61]; oscillatory power and the μ- and β-band components further modify these relations [62,63]. Membrane potential, recent activity, oscillatory state, neuromodulatory tone, and behavior all contribute, so no single variable captures brain state. Neuronavigation reduces geometric variability but cannot control these temporal fluctuations [12,64].

State also changes over longer timescales. Motor learning can alter subsequently induced plasticity [65], preconditioning with transcranial direct current stimulation can change responses to the same repetitive transcranial magnetic stimulation (rTMS) protocol [66], and GABAergic or glutamatergic drugs can modify intracortical measures without changing resting motor threshold [56,57,58]. These observations are compatible with homeostatic metaplasticity but are not deterministic across participants or protocols. Recent motor or cognitive activity, medication, prior sessions, and interblock intervals should therefore be documented and, when feasible, standardized.

## 6. Synaptic, Molecular, and Glial Mechanisms

### 6.1. LTP- and LTD-like Synaptic Plasticity

Persistent changes after repetitive TMS are commonly described as long-term potentiation (LTP)-like or long-term depression (LTD)-like plasticity. The qualifier “-like” is essential: human studies generally infer plasticity from sustained changes in MEPs, TMS-evoked EEG responses, or behavior rather than direct measurements of synaptic strength [8,18]. Paired associative stimulation demonstrated timing-dependent facilitation or suppression [67], and theta-burst stimulation produced directionally different average after-effects under specified motor-cortex conditions [68]. Memantine blocked both effects, supporting NMDA-receptor involvement [69], but NMDA dependence and persistent output changes do not prove canonical cellular LTP or LTD. Intrinsic excitability, inhibition, network synchronization, and spinal mechanisms may also contribute. Direct synaptic and molecular mechanisms are therefore evaluated below primarily as preclinical evidence that generates hypotheses for human nTMS [11].

Experimental preparations provide a more direct bridge to synaptic mechanisms. In mouse organotypic hippocampal cultures, 10 Hz repetitive magnetic stimulation produced persistent glutamatergic potentiation, spine remodeling, and NMDA-receptor-dependent AMPA-receptor changes [70,71]. A 600-pulse intermittent theta-burst stimulation (TBS) protocol likewise potentiated dentate-granule-cell synapses and induced coordinated spine remodeling lasting up to 24 h, with an orientation-dependent c-Fos response [72]. Multiscale modeling reproduced preferential proximal dendritic potentiation and predicted engagement of both proximal and distal compartments [73]. These rodent hippocampal preparations directly demonstrate changes in synaptic efficacy and structure, but they cannot establish the corresponding mechanism in the human neocortex or clinical nTMS.

### 6.2. Intracellular Signaling, Brain-Derived Neurotrophic Factor (BDNF), and Activity-Dependent Gene Expression

Calcium-dependent signaling provides one link from depolarization to persistent modification. NMDA-receptor and L-type-channel calcium entry activates calcium/calmodulin-dependent protein kinase II (CaMKII), cAMP response element-binding protein (CREB), and plasticity-related transcription, including brain-derived neurotrophic factor (BDNF) [74,75]. High-frequency magnetic stimulation increased CaMKII–CREB signaling and BDNF expression in Neuro-2a cells [75], while daily 5 Hz rTMS enhanced BDNF–tropomyosin receptor kinase B (TrkB) signaling in rat prefrontal cortex [76]. In organotypic cultures, disrupting BDNF–TrkB signaling prevented 10 Hz magnetic stimulation from potentiating excitatory transmission [77]. These results support a preclinical BDNF–TrkB mechanism, but peripheral BDNF concentrations do not quantify cortical signaling in humans.

Activity-dependent gene expression can extend plasticity beyond transient receptor activation. In rats, 1 Hz, 10 Hz, and intermittent TBS produced distinct regional patterns of immediate-early gene expression [78]. Anesthesia altered the direction of BDNF- and glutamate-receptor-related responses to repeated high-frequency rTMS [79], and transcriptomic profiling suggested a progression from immediate-early genes after one session to structural and growth-related programs after five sessions [80]. Gene induction nevertheless indicates cellular activation, not beneficial or durable plasticity, and interpretation depends on sample size, protocol, tissue-collection timing, brain state, and experimental preparation. Reproducing the physical input does not guarantee a reproducible transcriptional response.

### 6.3. Glial and Neuroimmune Contributions to Plasticity

Lasting plasticity also develops within a neuron–glia system. In cultured mouse astrocytes, low-intensity repetitive magnetic stimulation altered calcium-signaling, inflammatory, and adhesion pathways in a frequency- and intensity-dependent manner [81]. In ischemic rats, rTMS shifted microglial and cytokine profiles toward an anti-inflammatory phenotype [82]. In adult mice, low-intensity intermittent TBS increased the survival and maturation of new oligodendrocytes and enhanced myelination, whereas continuous TBS and 10 Hz stimulation did not [83].

These findings suggest that glial cells can translate repeated neuronal activity into homeostatic, trophic, inflammatory, and myelin-related adaptations. The evidence, however, comes predominantly from cell cultures, low-intensity stimulation, and animal models. It does not establish that clinical nTMS has direct anti-inflammatory or remyelinating effects in humans. Neuronavigation can reproduce the spatial distribution of the physical stimulus but cannot confer cell-type specificity within the exposed tissue.

## 7. From Local Cortical Activation to Network-Level Plasticity

### 7.1. Transsynaptic Propagation and Distributed Network Effects

The focality of the induced electric field should not be equated with the extent of the biological response. After local recruitment, activity can propagate through horizontal, commissural, association, and cortico-subcortical pathways. Early TMS–EEG showed spread from the left sensorimotor hand area to adjacent ipsilateral regions within 5–10 ms and to homologous contralateral areas within approximately 20 ms [84]. Concurrent TMS combined with positron emission tomography (TMS–PET) likewise detected blood-flow changes in connected regions beyond the localized target [85]. In a neuronavigated TMS–EEG study with individual diffusion and functional magnetic resonance imaging (fMRI), structural connectivity predicted propagation from default-mode and dorsal-attention network nodes better than resting-state functional connectivity [86]. A navigated cortical target is therefore an entry point into a network, not an isolated functional unit.

Propagation is not a passive continuation of the induced field. Macaque single-unit recordings found direct spiking effects within a region less than 2 mm in diameter [87], distinguishing local recruitment from synaptically mediated distributed responses. In humans, combined TMS–EEG and diffusion imaging linked callosal microstructure to interhemispheric propagation between homologous motor and prefrontal regions [88].

Not every distant response represents transsynaptic cortical activity. The coil click, scalp and cranial-muscle stimulation, somatosensory input, and motor reafference can all contribute to EEG and neuroimaging signals. Realistic multisensory sham stimulation can evoke complex EEG potentials resembling active TMS responses at early and late latencies [89]. Demonstrating network engagement therefore requires auditory masking, realistic sham and peripheral sensory controls, and preferably source-resolved recordings. Multimodal evidence is most convincing when electrophysiology, imaging, and connectivity measures provide convergent but nonredundant information about engagement of the intended network.

### 7.2. Oscillations, Functional Connectivity, and Brain-State Dependence

Brain state influences both local excitability and the propagation of stimulation through distributed networks. EEG-triggered motor-cortex TMS demonstrated phase-dependent differences in induced plasticity [61], with modulation of motor-evoked potentials most evident at lower stimulation intensities [90]. In a small neuronavigated TMS–EEG–fMRI study, higher prestimulus alpha power attenuated evoked hemodynamic responses across cortical and subcortical motor regions among participants with measurable motor-network reactivity [91]. Open-loop nTMS can therefore reproduce target location and coil geometry without controlling the physiological state that gates network propagation. Current evidence, however, comes mainly from healthy participants and motor-system paradigms, and a clinical advantage of state-dependent stimulation has not yet been established [12,64].

Repeated stimulation can modify network coupling in patterns not predicted by a simple inhibitory–excitatory frequency distinction. After left posterior inferior parietal stimulation, 20 Hz rTMS reduced correlations among default-mode cortical components, whereas 1 Hz stimulation increased target–hippocampal connectivity [92]. In a sham-controlled study, high-frequency left dorsolateral prefrontal cortex (DLPFC) stimulation increased anterior cingulate connectivity within one mesocorticolimbic network but not the other networks examined [93]. Network plasticity is therefore circuit-dependent and may redistribute rather than uniformly increase or decrease coupling. Neuronavigation reproduces engagement of a cortical node but does not determine the downstream response; fMRI connectivity remains an indirect, correlational measure.

## 8. Protocol-Dependent Mechanistic Signatures

### 8.1. Low- and High-Frequency rTMS

Conventional rTMS protocols are commonly classified by frequency. Early motor-cortex studies found that rapid-rate stimulation could facilitate or suppress corticospinal output depending on both frequency and intensity [94], while approximately 1 Hz stimulation often reduced and 20 Hz stimulation often increased MEP amplitude [95,96]. These observations motivated the shorthand association of low frequency with inhibitory or LTD-like effects and high frequency with facilitatory or LTP-like effects. MEP amplitude, however, reflects the net output of cortical, corticospinal, spinal, and peripheral elements rather than direct synaptic depression or potentiation. Frequency labels describe protocol classes, not guaranteed biological outcomes.

Frequency–response curves differ among participants, and increasing the pulse number from 240 to 1600 did not remove this variability [97]. In a sham-controlled repeated-session study, neither 1 Hz nor 10 Hz rTMS changed corticospinal excitability significantly at group level, and active-protocol reproducibility was poor [98]. These single-session motor-cortex findings do not determine clinical efficacy, but they show why frequency cannot be interpreted without intensity, pulse number, train structure, waveform, current direction, anatomy, and prestimulation state.

Neuronavigation reduces the spatial component of variability. In a crossover study of ten healthy participants, navigated 1 Hz rTMS produced more robust physiological and behavioral effects than non-navigated stimulation, consistent with improved reproduction of coil placement and orientation [99]. This small study does not establish a general superiority of navigation for all protocols, and spatial precision cannot eliminate biological variability. Mechanistic studies should report target definition, coil pose, stimulation intensity, complete temporal structure, positioning accuracy, and, when available, the modeled electric field and relevant state variables.

### 8.2. Theta-Burst Stimulation

Theta-burst stimulation (TBS) delivers patterned pulses over a much shorter period than conventional rTMS. The standard pattern consists of 50 Hz triplets repeated every 200 ms. Continuous TBS (cTBS) delivers 600 uninterrupted pulses over 40 s, whereas intermittent TBS (iTBS) applies 2 s trains every 10 s and delivers 600 pulses over 190 s. In the original motor-cortex experiments, iTBS increased MEP amplitudes for approximately 15 min, while cTBS suppressed them for nearly 60 min [68]. Memantine abolished both effects, supporting NMDA-receptor involvement [69]. Epidural recordings suggested partly distinct circuit effects: cTBS preferentially suppressed the early I1-wave, whereas iTBS facilitated later transsynaptically generated I-waves [100,101]. Temporal spacing can therefore produce different responses despite identical intraburst frequency and total pulse number [102,103].

Canonical TBS after-effects are not fixed. Increasing each protocol to 1200 pulses reversed the expected direction in one study [104], and voluntary contraction during or after stimulation also modified or reversed responses [105]. The Big TMS Data Collaboration pooled 430 healthy participants from 22 studies and identified baseline MEP amplitude, target muscle, time of day, stimulation device, and measurement timing as contributors to variability, with different effects for iTBS and cTBS [106]. Accordingly, iTBS and cTBS identify temporal stimulation patterns; “facilitatory” and “inhibitory” describe conditional average responses, not deterministic biological outcomes [102,103].

Neuronavigation reduces spatial and angular targeting error [4,107] but cannot by itself ensure reproducible TBS after-effects [106]. TBS remains a multidimensional intervention defined by intensity, pulse number, temporal spacing, coil orientation, electric-field distribution, prior activity, and brain state. Its short duration is a practical advantage, not evidence of mechanistic uniformity.

### 8.3. Paired Associative Stimulation

Paired associative stimulation (PAS) repeatedly couples two inputs at a defined interstimulus interval to induce timing-dependent associative plasticity. In its classical sensorimotor form, electrical stimulation of a peripheral nerve is paired with TMS over the contralateral primary motor cortex so that afferent input and TMS-evoked cortical activity converge in a selected temporal order. Depending on that interval and other protocol variables, corticospinal excitability may increase or decrease for tens of minutes; these changes are commonly described as long-term potentiation (LTP)-like or long-term depression (LTD)-like after-effects. TMS–EEG findings indicate that PAS also modulates cortical responses, but these measures remain indirect and do not constitute direct observation of synaptic LTP or LTD [108].

Cortico-cortical PAS (ccPAS) replaces the peripheral input with a conditioning TMS pulse delivered to one cortical area, followed at a defined interval by a second pulse to an anatomically or functionally connected area. Repeated pairing is intended to modify pathway-specific effective connectivity through mechanisms analogous to spike-timing-dependent plasticity. ccPAS has been extended from motor circuits to sensory, parietal, prefrontal, and other higher-order networks, although protocols and outcomes remain heterogeneous and evidence for clinical efficacy is limited [109,110].

Because the intended effect depends on the order, timing, and location of stimulation at two network nodes, ccPAS places particularly high demands on spatial and temporal reproducibility. Neuronavigation can improve the reproducibility of target localization, coil position, and orientation across pairings and sessions, especially outside the motor system, but it does not control conduction delays, baseline excitability, ongoing brain state, or a pathway’s susceptibility to plastic change [19,107,109]. PAS should therefore be regarded as a timing- and pathway-defined mechanistic probe rather than a deterministic means of inducing LTP or LTD.

## 9. Neuronavigation as a Mechanistic Control Layer

### 9.1. Spatial Reproducibility, Coil Orientation, and Target Engagement

Neuronavigation co-registers the head with structural imaging and tracks target location, coil position, orientation, and scalp distance in real time. This limits displacement- and rotation-related variation in the induced field and supports reproducible coil–brain geometry within and across sessions [19,111].

Technical gains have been quantified in specific datasets. In 42 participants (11,230 measurements), mean target deviation was 10.66 mm with Beam F3 cap placement and 0.30 mm with neuronavigation [4]. The cap method also showed greater angular and interoperator variability, and some markedly off-target placements delivered as little as 48.6% of the intended modeled field magnitude at the target [4]. The 0.30-mm value reflects tracked placement relative to registered coordinates, not total anatomical accuracy. A separate simulation-based analysis estimated overall coil–head coregistration accuracy at 2.2–3.6 mm and approximately 1°, depending on registration and model realism [107]. These are study-specific, not universal, performance estimates.

These gains reduce technical variance but do not guarantee equivalent cortical exposure or biological response. Reports should specify the navigation system, target-definition and registration methods, positional and angular tolerances, and management of head movement. Geometric accuracy is evidence of reproducible delivery, not target engagement or efficacy.

### 9.2. From Anatomical Navigation to Electric-Field-Informed Stimulation

Anatomical reproducibility does not imply equivalent intracranial exposure: coil-to-cortex distance, cortical folding, tissue geometry, and conductivity can alter the field despite identical scalp coordinates and stimulator settings [13]. Individualized head models therefore combine imaging, segmentation, coil pose, and numerical simulation to estimate field magnitude and direction [16,37,38].

Patient-specific simulations can prospectively optimize coil configuration for a prespecified field metric within a region of interest and adjust intensity toward a target cortical field strength rather than a fixed percentage of motor threshold [39,40,112]. Rapid algorithms can search millions of configurations within minutes, making this approach increasingly feasible [112].

Predictions remain sensitive to segmentation, assigned conductivities, coil representation, numerical resolution, and the chosen objective; total magnitude, directional components, spatial extent, and orientation may favor different configurations [37,40,43]. Moreover, an optimized field metric does not establish neuronal recruitment, which depends on morphology, state, and connectivity. Whether exposure equalization improves physiological reproducibility or outcomes remains uncertain. Electric-field-informed navigation advances biophysical precision but still requires biological validation [16].

## 10. Variability and Mechanistic Biomarkers

### 10.1. Sources of Inter- and Intraindividual Variability

Even with reproduced coil geometry, TMS responses vary across individuals and sessions because of technical delivery, anatomy and circuit organization, dynamic physiology, medication or behavioral context, and measurement. Stable factors include cortical anatomy, network architecture, genetic background, and current-direction-sensitive recruitment; dynamic factors include attention, arousal, recent activity, sleep pressure, circadian phase, and medication [18,113,114,115]. Neuronavigation addresses only part of the delivery variance [12,64].

Evidence supports contributions from both circuit recruitment and brain state. In 56 healthy participants, responses to excitatory and inhibitory TBS were associated with the latency of MEPs produced by anterior-to-posterior currents, suggesting that different interneuronal pathways contributed to response variability [113]. Repeated TMS–EEG during sustained wakefulness also showed changes in cortical excitability related to circadian phase and accumulated sleep pressure [114]. Protocol parameters interact as well: doubling standard TBS protocols reversed their expected effects [104], while changing cTBS intensity abolished or partly reversed modulation of corticospinal excitability [116].

Mechanistic studies should report programmed parameters, target definition, recorded coil pose and positioning error, outcome procedures, and modeled electric-field exposure when available. Recent activity, medication, sleep, and time of day should be controlled or documented. Table 1 links the major variance sources with practical controls.

### 10.2. Biomarkers of Target Engagement and Plasticity

Target engagement denotes a measurable response of the intended cortical region or network to stimulation, whereas plasticity requires a change that persists beyond the immediate perturbation. Although related, these concepts should not be treated as interchangeable.

Motor thresholds, MEP amplitudes, and stimulus–response curves characterize corticospinal excitability and output. Paired-pulse measures and the cortical silent period probe inhibitory and facilitatory interactions [117,118]. These measures remain indirect and depend on cortical recruitment, corticospinal and spinal excitability, peripheral conduction, recording quality, and protocol. Their utility in the motor cortex does not establish validity for nonmotor targets.

TMS–EEG records local and distributed evoked responses outside the motor system [28,84,119], but waveform, current direction, and auditory, somatosensory, muscular, ocular, and equipment-related artifacts can alter response morphology and amplitude [28,89,119]. Target-engagement inference therefore requires masking, realistic sham or sensory controls, artifact suppression, and standardized analysis [120,121,122].

EEG, functional MRI, and positron emission tomography can characterize oscillatory, hemodynamic, metabolic, and connectivity responses over time [84,85,123]. They are sensitive to acquisition, preprocessing, timing, and operational definitions and remain largely correlational. Convergence across controlled, nonredundant modalities is more informative than multiple readouts of the same artifact or physiological process [111].

Peripheral biochemical or neuroendocrine candidates remain indirect. A double-blind matched-pair study of 20 female volleyball players reported changes in salivary Orexin-A levels, attention, and reaction time after 10 Hz left DLPFC rTMS [124]. This neither validates Orexin-A as a marker of cortical engagement nor isolates a contribution from neuronavigation. Such measures require corroboration by central physiological, imaging, and functional outcomes.

No single measure is a validated universal biomarker of TMS-induced plasticity. Stronger inference requires a prespecified chain linking modeled exposure to a controlled local response, distributed engagement, and a durable behavioral or clinical outcome. Table 2 summarizes the inference and limitations for each measure.

## 11. Translational Implications

### 11.1. From Mechanistic Understanding to Individualized Stimulation

Mechanistic knowledge becomes clinically useful only when it informs what should be stimulated, how the stimulus should be delivered, and whether the intended neural system responded. This requires individualized target selection, electric-field-informed exposure planning, and physiological confirmation of engagement. Computational studies show that optimal coil position and orientation vary across regions and individuals [125]; identical device settings or scalp coordinates therefore cannot be assumed to produce equivalent cortical exposure.

Computational modeling may also help predict the motor hotspot and motor threshold before stimulation. In a proof-of-concept study of ten healthy participants, predicted hotspots were located, on average, 1.3 cm from experimentally determined sites, while modeled electric-field strength was associated with the measured motor threshold [126]. These findings support further development of individualized planning, but they do not justify replacing physiological measurements or established safety procedures.

Precision nTMS is better understood as a workflow than as an inherent property of a navigation system. It requires an individually defined target, planned and reproducible stimulus delivery, and evidence that the intended neural system was engaged. Accurate return to a scalp coordinate satisfies only part of this process and does not, by itself, demonstrate biological specificity.

### 11.2. Toward Adaptive and Closed-Loop nTMS

Most nTMS protocols remain open-loop: target location and stimulation parameters are selected in advance and remain unchanged despite ongoing fluctuations in cortical excitability. Closed-loop approaches instead use real-time physiological signals, most commonly EEG, to determine when a pulse should be delivered or how stimulation should be adapted to the current brain state.

In the healthy motor cortex, otherwise identical rTMS produced LTP-like increases in corticospinal excitability when pulses were triggered during a high-excitability phase of the sensorimotor μ-rhythm, but not during a low-excitability phase [61]. Early clinical evidence remains preliminary. In a single-session study involving 17 patients with antidepressant-resistant major depressive disorder, real-time EEG triggering of left DLPFC stimulation was successfully achieved in 15 participants and produced phase-dependent neurophysiological effects. However, the study was designed to assess feasibility and immediate target modulation rather than therapeutic efficacy [127].

Neuronavigation and closed-loop control address complementary dimensions: navigation constrains where the field is delivered, whereas physiological feedback may refine when it is delivered and in which physiological state. A near-term experimental model could combine a stable, electric-field-informed target with brain-state-dependent timing and periodic reassessment of engagement. Translation requires validated input signals, artifact control, low-latency processing, reproducible algorithms, and explicit safety constraints [128]. No current evidence establishes general clinical superiority of closed-loop over conventional approaches; adaptive nTMS should remain an experimental strategy pending prospective comparative trials [12,64].

## 12. Limitations and Future Research Directions

### 12.1. Methodological and Mechanistic Limitations

Several limitations constrain the mechanistic interpretation of nTMS. Most cellular and molecular evidence derives from TMS experiments that were not designed to isolate the contribution of neuronavigation, while direct nTMS-specific mechanistic data remain limited. Its application to nTMS is therefore inferential and rests on the premise that neuronavigation modifies the spatial and orientational properties of stimulus delivery without creating a distinct downstream biological mechanism.

The evidence considered in this review spans in vitro experiments, animal models, human motor-cortex studies, and clinical investigations using heterogeneous coils, waveforms, targets, intensities, and outcome measures. Extrapolation from acute cellular responses to durable plasticity in human brain networks consequently remains uncertain. Human studies frequently include small samples of healthy participants, focus predominantly on the primary motor cortex, and use short-term changes in motor-evoked potentials as surrogate outcomes. A large pooled analysis of theta-burst stimulation showed that participant characteristics, baseline measurements, and methodological choices contribute substantially to response variability [106]. TMS–EEG and neuroimaging measures are additionally affected by sensory contamination, preprocessing decisions, analytic flexibility, and incomplete interlaboratory reproducibility [129]. Electric-field models depend on MRI segmentation, assumed tissue conductivities, coil representations, and numerical implementation. They estimate physical exposure but do not directly identify the neuronal elements that were recruited [16,103,120,121,130].

Greater spatial precision does not necessarily produce stronger or more durable clinical effects when disease-related network reorganization, medication, behavior, and fluctuating brain state remain uncontrolled. Several links between electric-field delivery and functional plasticity remain plausible rather than conclusively demonstrated. This narrative review used a targeted, iterative search without prospective registration, duplicate screening, a formal study-level risk-of-bias instrument, or quantitative synthesis. Records examined during the original development were not prospectively logged, so the documented rerun improves reproducibility but cannot create a retrospective systematic-review cohort or item-level exclusion flow. Study selection and evidence weighting therefore remain susceptible to selection and interpretive bias. The proposed framework is an integrative, hypothesis-generating structure, not a validated classification, taxonomy, or causal model.

### 12.2. Priorities for Future Research

Future studies should report enough information to reconstruct the delivered stimulus and its modeled exposure. At minimum, reports should specify the intended anatomical target, target-definition method, actual recorded coil pose, positional and angular targeting error, coil-to-cortex distance, coil and stimulator characteristics, waveform, intensity, pulse number and timing, motor-threshold procedure when used, and the individualized field metric and modeling pipeline when available [131]. Medication, behavioral or physiological state, and outcome timing should also be documented. These data distinguish intended delivery from achieved geometry and allow between-study differences to be interpreted [16,111].

Preregistered multicenter studies should directly compare standard scalp-based targeting and motor-threshold dosing with neuronavigated, electric-field-informed, and biomarker-guided strategies [4]. Such studies should incorporate credible sham conditions, blinded outcome assessment, and clinically meaningful endpoints. They should also extend beyond small studies of healthy motor cortex to disease-specific populations and nonmotor networks. Ideally, electric-field estimates, local electrophysiological responses, network-level measurements, and behavioral or clinical outcomes would be acquired in the same participants at multiple time points. This design would allow investigators to test whether early target engagement mediates subsequent network reorganization and durable functional change.

Candidate biomarkers should be externally validated, demonstrate prospective predictive value rather than merely post hoc associations, and provide reproducible criteria for meaningful target engagement [119,128]. Shared datasets, harmonized preprocessing pipelines, and transparent reporting of negative findings will likewise be necessary to distinguish robust mechanisms from laboratory-specific effects [120,121,122,129].

The objective should not be to maximize technological complexity or personalization for its own sake, but to determine which additional layer of precision produces a reproducible biological and clinical benefit. A more accurate electric-field map matters only if it ultimately contributes to a better patient outcome.

## 13. Conclusions

Precision nTMS is a chain linking the intended target and recorded coil pose to individualized intracranial exposure, neural and network engagement, and durable functional or clinical benefit. Neuronavigation improves geometric precision, and modeling estimates biophysical exposure; neither alone establishes biological precision or efficacy. Each link requires independent validation. Prospective comparative studies should therefore test whether electric-field-informed planning, multimodal target-engagement measures obtained under controlled conditions, or adaptive stimulation produce reproducible biological effects and clinically meaningful outcomes.

## Figures and Tables

**Table 1:** Sources of inter- and intraindividual variability in neuronavigated transcranial magnetic stimulation and strategies for their control.

Domain	Representative Sources of Variability	Potential Effect on Exposure or Response	Control, Mitigation, and Reporting
Device and protocol	Coil design; pulse waveform and width; current direction; intensity; frequency; pulse number; train structure; intertrain and intersession intervals; stimulator characteristics [17,25,26,27,28,94,95,96,97,98,99,100,101,104,105,106].	Changes the electric-field waveform and distribution, the neuronal populations recruited, and the direction or durability of after-effects.	Use calibrated equipment and fully report coil, stimulator, waveform, current direction, intensity, pulse pattern, total pulse count, and temporal structure.
Spatial delivery	Target definition; coil position, rotation, tilt, and scalp distance; coregistration error; head and tracker movement; operator variability [4,23,24,107,112].	Shifts electric-field location, magnitude, and direction and may recruit different cortical elements or pathways.	Use MRI-based neuronavigation, real-time pose tracking, predefined positional and angular tolerances, motion checks, and intersession reproduction of coil geometry.
Anatomy and physical exposure	Coil-to-cortex distance; cortical folding; skull and cerebrospinal fluid geometry; tissue conductivity; atrophy, lesions, edema, cavities, or cranial reconstruction [13,14,29,30,31,32,33,34,35,36,37,38,39,40,41].	Produces subject- and region-specific differences in electric-field strength, direction, focality, and exposed tissue volume despite identical device settings.	Use individual structural imaging and, when relevant, lesion-aware electric-field modeling; report segmentation, conductivities, coil model, field component, region of interest, and exposure metric.
Neural and circuit properties	Axonal geometry and myelination; cytoarchitecture; current-direction sensitivity; balance of excitatory and inhibitory circuits; network architecture [3,42,43,44,45,46,47,48,49,50,51,52,53,54,55,56,57,58,59,60,61,62,63,100,101,113].	Determines which neural elements and microcircuits reach threshold and whether nominally similar stimulation engages comparable pathways.	Maintain coil orientation and induced-current direction; combine physical targeting with physiological mapping or target-engagement measures. These factors cannot be fully controlled by neuronavigation.
Dynamic physiological state	Oscillatory phase and power; arousal; attention; sleep pressure; circadian phase; recent activity or learning; prior stimulation [18,61,62,63,65,66,90,91,104,105,106,114,115,116].	Alters momentary excitability, recruitment threshold, network propagation, and the probability or direction of induced plasticity.	Standardize or document time of day, sleep, recent activity, and prestimulation state; use repeated baseline measurements. Brain-state-triggered stimulation remains experimental.
Medication and behavioral context	GABAergic, glutamatergic, and other centrally acting medication; voluntary contraction; concurrent task; behavioral engagement [56,57,58,65,79,105,115].	Modifies intracortical inhibition, facilitation, metaplasticity, and measured corticospinal or network responses.	Prespecify medication rules and behavioral conditions; document dose and timing; keep muscle activation and task context consistent across sessions.
Measurement and analysis	Baseline variability; target muscle and recording quality; measurement timing; sensory contamination; sham design; preprocessing and analytic choices [84,89,106,114].	May create, obscure, or reverse an apparent stimulation effect without a corresponding difference in the underlying biological response.	Preregister outcomes and time points; standardize acquisition and preprocessing; use credible sensory controls, blinded assessment, quality criteria, and longitudinal or multimodal confirmation.

Abbreviations: MRI, magnetic resonance imaging; nTMS, neuronavigated transcranial magnetic stimulation; TBS, theta-burst stimulation; TMS, transcranial magnetic stimulation.

**Table 2:** Candidate measures of target engagement and plasticity in precision nTMS.

Candidate Measure	Level of Inference	Principal Strength	Main Limitation and Appropriate Interpretation
Individualized electric-field model	Physical exposure within a predefined cortical region [36,37,38,39,40,41,107,112].	Links anatomy and recorded coil pose to an individualized estimate of field magnitude, direction, and spatial distribution.	Depends on segmentation, conductivity assumptions, coil representation, numerical implementation, and selected field metric. It is an exposure estimate, not direct evidence of neuronal activation.
Motor threshold, MEP amplitude, and input–output curve	Threshold and gain of corticospinal output in the motor system [49,50,51,52,53,54,100,106,117].	Widely available, temporally precise, and suitable for repeated assessment of motor-cortex responses.	Influenced by cortical, corticospinal, spinal, peripheral, and recording factors; limited generalizability to nonmotor targets. It should not be interpreted as a direct measure of local membrane depolarization.
Paired-pulse measures and cortical silent period	Systems-level inhibitory and facilitatory motor-cortical interactions [55,56,57,58,59,60,118].	Provides complementary circuit-sensitive phenotypes with established pharmacological responsiveness.	Results depend on pulse intensities, current direction, test MEP, and overlapping circuits. SICI, ICF, LICI, or silent-period duration are not quantitative assays of a single receptor or neurotransmitter.
TMS–EEG	Local cortical reactivity, oscillatory state, and propagation to connected regions [28,61,62,63,84,89,90,119].	Extends physiological assessment beyond the motor system and supports millisecond-scale measurement of local and distributed responses.	Auditory, somatosensory, muscular, ocular, and equipment-related components can dominate the signal. Target engagement requires appropriate masking, sensory controls, artifact suppression, and standardized analysis.
fMRI, PET, and connectivity measures	Hemodynamic, metabolic, and network-level consequences of stimulation [85,86,91,92,93,123].	Characterizes distributed engagement and longitudinal network reorganization beyond the stimulated cortical entry point.	Predominantly correlational and sensitive to acquisition, preprocessing, timing, and connectivity definitions. A change in connectivity does not independently establish synaptic plasticity or causal clinical relevance.
Molecular and glial mechanisms	Activity-dependent signaling, gene expression, neurotrophic pathways, and glial responses [74,75,76,77,78,79,80,81,82,83].	Provides biologically plausible links between patterned magnetic stimulation and persistent cellular adaptation in preparations that permit direct mechanistic measurement.	Evidence is predominantly preclinical and often uses low-intensity stimulation or nonhuman tissue. These findings define mechanistic hypotheses, not validated biomarkers of human nTMS.
Peripheral biochemical and neuroendocrine measures	Systemic or neuroendocrine correlates measured alongside stimulation and functional outcomes [124].	May provide repeatable, minimally invasive complementary information about systemic responses and support multimodal longitudinal designs.	Peripheral concentrations do not establish local cortical concentration, receptor engagement, or a causal neural mechanism. These measures remain exploratory and require central physiological or imaging corroboration.
Behavioral and clinical outcomes	Durable functional relevance of the induced response [9,10,18].	Provides the endpoint that ultimately determines whether additional targeting or monitoring complexity has practical value.	Usually temporally remote and mechanistically nonspecific. It validates clinical relevance but cannot by itself identify where the causal chain succeeded or failed.

Abbreviations: BDNF, brain-derived neurotrophic factor; EEG, electroencephalography; fMRI, functional magnetic resonance imaging; ICF, intracortical facilitation; LICI, long-interval intracortical inhibition; MEP, motor-evoked potential; nTMS, neuronavigated transcranial magnetic stimulation; PET, positron emission tomography; SICI, short-interval intracortical inhibition; TMS, transcranial magnetic stimulation.

## Data Availability

No new data were created or analyzed in this study. Data sharing is not applicable to this article.

## Supplementary Materials

The following supporting information can be downloaded at https://www.mdpi.com/article/10.3390/brainsci16090901/s1, Table S1: Reproducible PubMed search strategies and retrieval counts.

## Abbreviations

The following abbreviations are used in this manuscript:

BDNF	brain-derived neurotrophic factor
ccPAS	cortico-cortical paired associative stimulation
cTBS	continuous theta-burst stimulation
DLPFC	dorsolateral prefrontal cortex
EEG	electroencephalography
E-field	electric field
fMRI	functional magnetic resonance imaging
ICF	intracortical facilitation
iTBS	intermittent theta-burst stimulation
LICI	long-interval intracortical inhibition
LTD	long-term depression
LTP	long-term potentiation
MEP	motor-evoked potential
MRI	magnetic resonance imaging
nTMS	neuronavigated transcranial magnetic stimulation
PAS	paired associative stimulation
PET	positron emission tomography
rTMS	repetitive transcranial magnetic stimulation
SANRA	Scale for the Assessment of Narrative Review Articles
SICI	short-interval intracortical inhibition
TBS	theta-burst stimulation
TMS	transcranial magnetic stimulation
TMS–EEG	concurrent transcranial magnetic stimulation and electroencephalography
