# Supplementary material for: Precision in Delivery, Variability in Response: A Multiscale Mechanistic Framework for Neuronavigated Transcranial Magnetic Stimulation
Source: Brain Sci. 2026 Aug 23;16(9):901. doi: 10.3390/brainsci16090901 (PMC13604321; doi:10.3390/brainsci16090901)
Supplement: Supplementary file 1 [file brainsci-16-00901-s001.zip › brainsci-4513484-supplementary.pdf]

Supplementary Materials

Precision in Delivery, Variability in Response: A Multiscale Mechanistic Framework for Neuronavigated Transcranial Magnetic Stimulation

Marcin Karol Setlak, Bartłomiej Błaszczyk, Maciej Wojtacha, and Adam Rudnik

Table S1. Reproducible PubMed search strategies and retrieval counts.

The domain-specific searches were rerun in PubMed on 17 August 2026. The publication-date window was 1 January 1985 to 31 July 2026, and results were limited to English-language records. No study-design filter was applied.

Search/domain	Complete PubMed query	Records retrieved
**Q1**<br>**Biophysics and electric-field distribution**	("transcranial magnetic stimulation"[Title/Abstract] OR "repetitive transcranial magnetic stimulation"[Title/Abstract] OR "repetitive TMS"[Title/Abstract] OR rTMS[Title/Abstract] OR "theta-burst stimulation"[Title/Abstract] OR "theta burst stimulation"[Title/Abstract] OR "neuronavigated TMS"[Title/Abstract] OR "navigated TMS"[Title/Abstract]) AND ((("electric field"[Title/Abstract] OR "electric-field"[Title/Abstract]) AND (model*[Title/Abstract] OR simulat*[Title/Abstract] OR coil[Title/Abstract] OR cortical[Title/Abstract])) OR "coil orientation"[Title/Abstract] OR "coil-to-cortex distance"[Title/Abstract] OR "current direction"[Title/Abstract] OR "pulse waveform"[Title/Abstract]) AND english[Language] AND ("1985/01/01"[Date - Publication] : "2026/07/31"[Date - Publication])	607
**Q2**<br>**Neural recruitment and local microcircuits**	("transcranial magnetic stimulation"[Title/Abstract] OR "repetitive transcranial magnetic stimulation"[Title/Abstract] OR "repetitive TMS"[Title/Abstract] OR rTMS[Title/Abstract] OR "theta-burst stimulation"[Title/Abstract] OR "theta burst stimulation"[Title/Abstract] OR "neuronavigated TMS"[Title/Abstract] OR "navigated TMS"[Title/Abstract]) AND ("neural recruitment"[Title/Abstract] OR "neuronal recruitment"[Title/Abstract] OR "cortical recruitment"[Title/Abstract] OR I-wave[Title/Abstract] OR I-waves[Title/Abstract] OR ((paired-pulse[Title/Abstract] OR "paired pulse"[Title/Abstract]) AND (SICI[Title/Abstract] OR ICF[Title/Abstract] OR LICI[Title/Abstract] OR intracortical[Title/Abstract])) OR ("state-dependent"[Title/Abstract] AND excitability[Title/Abstract])) AND english[Language] AND ("1985/01/01"[Date - Publication] : "2026/07/31"[Date - Publication])	1 004
**Q3**<br>**Synaptic, molecular, and glial mechanisms**	("transcranial magnetic stimulation"[Title/Abstract] OR "repetitive transcranial magnetic stimulation"[Title/Abstract] OR "repetitive TMS"[Title/Abstract] OR rTMS[Title/Abstract] OR "theta-burst stimulation"[Title/Abstract] OR "theta burst stimulation"[Title/Abstract] OR "neuronavigated TMS"[Title/Abstract] OR "navigated TMS"[Title/Abstract]) AND ("synaptic plasticity"[Title/Abstract] OR ("long-term potentiation"[Title/Abstract] AND plasticity[Title/Abstract]) OR ("long-term depression"[Title/Abstract] AND plasticity[Title/Abstract]) OR (BDNF[Title/Abstract] AND (TrkB[Title/Abstract] OR synaptic[Title/Abstract] OR plasticity[Title/Abstract])) OR (CaMKII[Title/Abstract] AND plasticity[Title/Abstract]) OR ("gene expression"[Title/Abstract] AND plasticity[Title/Abstract]) OR transcriptom*[Title/Abstract] OR astrocyt*[Title/Abstract] OR microglia*[Title/Abstract] OR oligodendrocyt*[Title/Abstract]) AND english[Language] AND ("1985/01/01"[Date - Publication] : "2026/07/31"[Date - Publication])	1 303

Table S1. Continued.

Search/domain	Complete PubMed query	Records retrieved
**Q4**<br>**Network effects, TMS–EEG, and neuroimaging**	("transcranial magnetic stimulation"[Title/Abstract] OR "repetitive transcranial magnetic stimulation"[Title/Abstract] OR "repetitive TMS"[Title/Abstract] OR rTMS[Title/Abstract] OR "theta-burst stimulation"[Title/Abstract] OR "theta burst stimulation"[Title/Abstract] OR "neuronavigated TMS"[Title/Abstract] OR "navigated TMS"[Title/Abstract]) AND (TMS-EEG[Title/Abstract] OR ("functional connectivity"[Title/Abstract] AND (fMRI[Title/Abstract] OR EEG[Title/Abstract] OR PET[Title/Abstract])) OR "network propagation"[Title/Abstract] OR "transsynaptic propagation"[Title/Abstract] OR (oscillat*[Title/Abstract] AND connectiv*[Title/Abstract]) OR ("brain state"[Title/Abstract] AND network*[Title/Abstract])) AND english[Language] AND ("1985/01/01"[Date - Publication] : "2026/07/31"[Date - Publication])	1 227
**Q5**<br>**Neuronavigation, targeting, and individualized modeling**	("transcranial magnetic stimulation"[Title/Abstract] OR "repetitive transcranial magnetic stimulation"[Title/Abstract] OR "repetitive TMS"[Title/Abstract] OR rTMS[Title/Abstract] OR "theta-burst stimulation"[Title/Abstract] OR "theta burst stimulation"[Title/Abstract] OR "neuronavigated TMS"[Title/Abstract] OR "navigated TMS"[Title/Abstract]) AND (neuronavigat*[Title/Abstract] OR "navigated TMS"[Title/Abstract] OR ("coil position"[Title/Abstract] AND reproducib*[Title/Abstract]) OR (("target localization"[Title/Abstract] OR "target localisation"[Title/Abstract]) AND TMS[Title/Abstract]) OR (("electric field modeling"[Title/Abstract] OR "electric field modelling"[Title/Abstract] OR "electric-field modeling"[Title/Abstract] OR "electric-field modelling"[Title/Abstract]) AND (individualized[Title/Abstract] OR individualised[Title/Abstract]))) AND english[Language] AND ("1985/01/01"[Date - Publication] : "2026/07/31"[Date - Publication])	771
**Q6**<br>**Variability, biomarkers, and adaptive stimulation**	("transcranial magnetic stimulation"[Title/Abstract] OR "repetitive transcranial magnetic stimulation"[Title/Abstract] OR "repetitive TMS"[Title/Abstract] OR rTMS[Title/Abstract] OR "theta-burst stimulation"[Title/Abstract] OR "theta burst stimulation"[Title/Abstract] OR "neuronavigated TMS"[Title/Abstract] OR "navigated TMS"[Title/Abstract]) AND ("target engagement"[Title/Abstract] OR biomarker*[Title/Abstract] OR closed-loop[Title/Abstract] OR "closed loop"[Title/Abstract] OR "EEG-triggered"[Title/Abstract] OR ("response variability"[Title/Abstract] AND (interindividual[Title/Abstract] OR intraindividual[Title/Abstract] OR reproducib*[Title/Abstract])) OR (adaptive[Title/Abstract] AND stimulation[Title/Abstract]) OR ((individualized[Title/Abstract] OR individualised[Title/Abstract]) AND dosimetry[Title/Abstract])) AND english[Language] AND ("1985/01/01"[Date - Publication] : "2026/07/31"[Date - Publication])	1 518

Notes: The six queries returned 6430 records in total before cross-query deduplication and 5617 unique PubMed identifiers. Counts document the search rerun and may change with subsequent PubMed indexing. Google Scholar and backward/forward citation tracking were used as complementary discovery methods; their dynamic rankings and result counts were not treated as reproducible countable search sets. The database outputs served as candidate pools for a purposive narrative synthesis and should not be interpreted as a prospectively logged systematic-review screening cohort.

Abbreviations: BDNF, brain-derived neurotrophic factor; EEG, electroencephalography; fMRI, functional magnetic resonance imaging; ICF, intracortical facilitation; LICI, long-interval intracortical inhibition; PET, positron emission tomography; rTMS, repetitive transcranial magnetic stimulation; SICI, short-interval intracortical inhibition; TMS, transcranial magnetic stimulation; TrkB, tropomyosin receptor kinase B.
